# Antiviral Drugs in HIV and Cardiovascular Disease: Mechanistic Insights and Clinical Implications

**DOI:** 10.3390/ph19020205

**Published:** 2026-01-25

**Authors:** Helal F. Hetta, Fawaz E. Alanazi, Hanan Alshareef, Saleh F. Alqifari, Salwa Qasim Bukhari, Mousa Aodh Albalwi, Zinab Alatawi, Asma Malwi Alshahrani, Eman M. Shorog, Ali M. Atoom, Abdelhakim A. Abdelrahman, Abdulrahman K. Ahmed, Yasmin N. Ramadan, Reem Sayad

**Affiliations:** 1 Division of Microbiology, Immunology and Biotechnology, Department of Natural Products and Alternative Medicine, Faculty of Pharmacy, University of Tabuk, Tabuk 71491, Saudi Arabia; 2 Department of Pharmacology and Toxicology, Faculty of Pharmacy, University of Tabuk, Tabuk 71491, Saudi Arabia; falanazi@ut.edu.sa; 3 Department of Pharmacy Practice, Faculty of Pharmacy, University of Tabuk, Tabuk 71491, Saudi Arabia; halsharef@ut.edu.sa (H.A.); salqifari@ut.edu.sa (S.F.A.); 4 Department of Diagnostic Radiology, Faculty of Medicine, University of Tabuk, Tabuk 71491, Saudi Arabia; s.bukhari@ut.edu.sa; 5 Department of Internal Medicine, Faculty of Medicine, University of Tabuk, Tabuk 71491, Saudi Arabia; mousa.albalwi@ut.edu.sa; 6 Department of Family and Community Medicine, Faculty of Medicine, University of Tabuk, Tabuk 47512, Saudi Arabia; zalatawi@ut.edu.sa; 7 Clinical Pharmacy Department, College of Pharmacy, Shaqra University, Shaqra 11911, Saudi Arabia; a.alshahrani@su.edu.sa; 8 Department of Clinical Pharmacy, College of Pharmacy, King Khalid University, Abha 62521, Saudi Arabia; eshorog@kku.edu.sa; 9 Faculty of Allied Medical Sciences, Hourani Center for Applied Scientific Research, Al-Ahliyya Amman University, Amman 19111, Jordan; a.atoom@ammanu.edu.jo; 10 King’s College Hospital, Denmark Hill, London SE5 9RS, UK; hakim.abdelrahman@nhs.net; 11 Emergency Medicine Unit, Department of Anaethesia and Intensive Care, Faculty of Medicine, Assiut University, Assiut 71515, Egypt; abdulrahmankhalifan7100@gmail.com; 12 Department of Microbiology and Immunology, Faculty of Pharmacy, Assiut University, Assiut 71515, Egypt; yasmine_mohamed@pharm.aun.edu.eg; 13 Department of Histology, Faculty of Medicine, Assiut University, Assiut 71515, Egypt; reem.17289806@med.aun.edu.eg

**Keywords:** HIV, cardiovascular diseases in HIV, antiretroviral therapy (ART), dyslipidemia

## Abstract

Cardiovascular disease (CVD) is increasingly recognized as a significant comorbidity in people living with HIV (PWH), contributing to increased morbidity and mortality. Epidemiological studies indicate that PWH have a 1.2–2-fold higher risk of myocardial infarction (MI) and other CVD events compared to HIV-negative individuals. While the mechanisms underlying HIV-associated CVD are not fully understood, they are likely to include a combination of cardiovascular-related adverse effects of HIV medications, vascular dysfunction caused by HIV-induced monocyte activation, and cytokine secretion, in addition to existing comorbidities and lifestyle choices. This comprehensive review examines the complex relationship between HIV infection and CVD, highlighting key pathophysiological mechanisms such as chronic immune activation, inflammation, endothelial dysfunction, and the role of antiretroviral therapy (ART) in promoting cardiovascular risk. Alongside conventional risk factors such as smoking, hypertension, and dyslipidemia, HIV-specific elements, especially metabolic abnormalities associated with ART, significantly contribute to the development of CVD. Prevention strategies are crucial, focusing on the early identification and management of cardiovascular risk factors as well as optimizing ART regimens to minimize adverse metabolic effects. Clinical guidelines now recommend routine cardiovascular risk assessment in PWH, emphasizing aggressive management tailored to their unique health profiles. However, challenges exist in fully understanding the cardiovascular outcomes in this population. Future research directions include exploring the role of inflammation-modulating therapies and refining sustainable prevention strategies to mitigate the growing burden of CVD in PWH.

## 1. Introduction

In 2023, 39.9 million patients were living with the Human Immunodeficiency Virus (HIV). Most of them are adults aged 15 years or older, 38.6 million. Children aged 0 to 14 years form a small proportion of all patients with HIV, 1.4 million. Women and girls constituted 53% of the total patients with HIV (PWH). In 2023, 86% (ranging from 69% to 98%) of individuals living with HIV were aware of their HIV status. Moreover, around 5.4 million individuals remained unaware of their HIV status [1].

HIV is a retrovirus from the genus Lentivirus that targets human T-cells [2,3]. It can use reverse transcription to convert its RNA genome into DNA. The DNA integrates into the genome of the host cell, promoting the virus’s growth and survival inside the human body. Chronic infection mainly affecting CD4^+^ T-cells is characterized by increasing lymphocyte death. Acquired immunodeficiency syndrome (AIDS) is a disorder characterized by specific symptoms resulting from immune system failure due to a reduced T-cell count [4].

PWH persistently exhibits an increased prevalence of cardiovascular disease (CVD), encompassing systolic heart failure (HF), atherosclerotic heart disease, and diastolic HF, compared to the general population [5,6,7,8,9]. Several mechanisms contributing to heightened atherogenesis and HF have been suggested, including elevated systemic inflammation, impaired autophagy, oxidative stress, direct impacts of viral proteins, inflammasome activation, and endoplasmic reticulum stress [10,11]. Observational studies have identified HIV viremia, immunosuppression, and HIV-associated dyslipidemia as variables that may predispose people with HIV to CVD [5,7,8,12,13,14,15].

Access to life-sustaining combination ART enables individuals with HIV to live longer, thereby elevating their risk for age-related illnesses, including CVD. According to the ATHENA cohort, 78% of PWH will be diagnosed with CVD, and the median age on ART is expected to rise from 43.9 years in 2010 to 56.5 years by 2030 [16]. Compared with HIV-uninfected individuals, people living with HIV have a higher relative risk of myocardial infarction—approximately 1.7-fold (RR~1.7) based on pooled estimates from recent systematic reviews and meta-analyses [17]. For both type 1 and type 2 acute MI events, the higher RR persists in people with viral suppression and may be more prominent in women than in men when it comes to coronary heart disease (CHD) [18]. Despite reduced absolute incidence of acute MI in those with fewer risk factors, the RR of acute MI remains elevated in PWH, including those in optimal heart health [19]. Ischaemic stroke constitutes over 80% of all strokes in individuals with HIV, with the remainder attributed to hemorrhagic stroke [20].

So, we performed this study to elucidate the pathophysiology, risk factors in PWH, and clinical guidelines for the prevention and management of these cases.

## 2. Pathophysiology of HIV and Its Association with Cardiovascular Diseases

**Figure 1:**
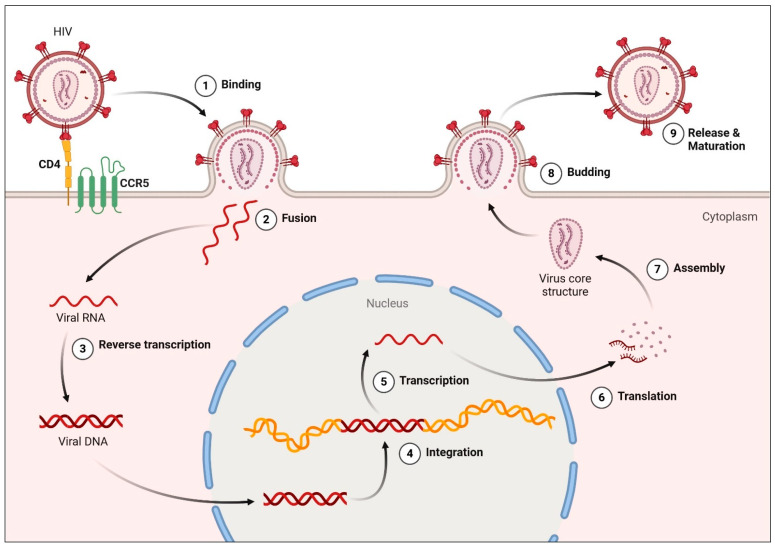
HIV Replication Cycle. Step 1 involves engagement between the viral envelope glycoprotein gp120 and the host CD4 receptor, followed by viral attachment, fusion, and entry into the host cell. In step 3, reverse transcriptase converts viral RNA into double-stranded DNA, a process targeted by nucleoside/nucleotide reverse transcriptase inhibitors (NRTIs) and non-nucleoside reverse transcriptase inhibitors (NNRTIs), which block viral DNA synthesis and prevent integration. The resulting viral DNA is integrated into the host genome, where transcription and translation produce viral RNA and proteins. Accumulation of viral products ultimately compromises host cell function, leading to depletion of CD4^+^ T-cells. Created in BioRender. Hetta, H. (2026) https://BioRender.com/yjca9s9 (accessed on 22 January 2026).

HIV undergoes multiple critical phases upon entering and persisting within a host organism. The host cell initially interacts with gp120, the viral envelope glycoprotein, via the CD4^+^ receptor and a chemokine co-receptor (CCR5 or CXCR4). This interaction facilitates viral attachment, fusion, and entry into the host cell. Reverse transcriptase, integrase, protease, and other enzymes are among the several enzymes that are released into the host cell’s cytoplasm during this process. By transforming the viral RNA genome into double-stranded DNA, reverse transcriptase helps integrate viral DNA into the host genome. This integration is an essential tactic the virus employs to escape the human immune system. Viral RNA and proteins are generated via transcription and translation of the integrated provirus. These are then released from the cell by budding. Immune cells inevitably disappear as the host cell’s function gradually declines. When the host’s immune system is significantly weakened, acquired immunodeficiency syndrome (AIDS) emerges, characterized by opportunistic infections and increased cancer susceptibility [21,22] (Figure 1).

HIV infection disrupts gut mucosal integrity, leading to microbial translocation and systemic exposure to microbial products. Dysbiosis in the gut microbiome contributes to elevated circulating levels of metabolites such as trimethylamine N-oxide (TMAO), which has been linked to endothelial dysfunction, inflammation, and accelerated atherogenesis in both the general population and PWH. These findings suggest that gut-derived metabolites may serve as mechanistic mediators connecting HIV-induced immune activation with cardiovascular risk. Recent studies found that higher plasma levels of the gut microbiome–related metabolite trimethylamine-N-oxide (TMAO) were associated with greater progression of carotid artery atherosclerosis in people living with HIV, with positive correlations to biomarkers of monocyte activation and inflammation (e.g., sCD14, sCD163) [23]. Studies have investigated TMAO in relation to inflammation, microbial dysbiosis, and immune activation in HIV cohorts, reflecting the complex relationship between gut microbiota–derived metabolites and systemic immune responses in HIV infection [24]. The microbiota-dependent metabolite TMAO was found to correlate with markers of monocyte activation and microbial translocation in untreated HIV-infected individuals, suggesting a possible link between microbial products and cardiovascular risk modulators in HIV [25].

**Figure 2:**
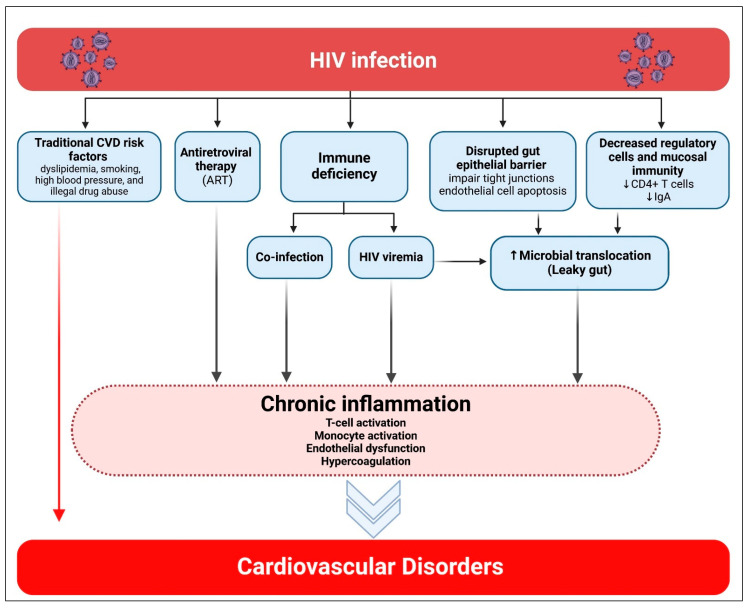
Association of HIV infection with cardiovascular diseases. Created in BioRender. Hetta, H. (2026) https://BioRender.com/opk308x (accessed on 22 January 2026).

Mechanisms are anticipated to vary by type and subtype of CVD as well as by geographical region, influenced by factors such as availability to ART, lifestyle, endemic comorbidities, and genetic predispositions [26]. However, among PWH across various regions, two prevalent factors contributing to CVD risk are identified (Figure 2). The first factor is increased systemic immunological activation, which has theoretical significance in the pathophysiology of both CHD, MI, and HF. HIV represents a paradoxical condition of immunological suppression alongside increased systemic immune activation [27]. ART effectively suppresses viremia; nevertheless, contemporary ART inadequately reduces systemic immune activation and vascular inflammation. In PWH, indicators of systemic monocyte activation have been associated with arterial inflammation, which catalyzes both vascular and cardiac disease. A second significant contributor to CVD risk among PWH is metabolic dysregulation, potentially linked to HIV infection, HIV-related immunological activation, and/or the administration of specific ART [28,29]. According to recent findings, immune cells and adipocytes residing in adipose tissue play a role in the immune response to HIV infection [30,31]. Adipose tissue may serve as a reservoir for HIV in virologically suppressed persons because it contains infected CD4^+^ T cells and macrophages [31,32]. Furthermore, even in the face of viral suppression, HIV proteins are still present in the blood and adipose tissue. Research conducted in vitro suggests that adipocytes exposed to HIV proteins produce proinflammatory mediators [31,33]. HIV results in metabolic inefficiency and subcutaneous adipose tissue fibrosis, which produce ectopic fat redistribution to the visceral area. Visceral fat buildup increases the risk of CVD and causes insulin resistance [28]. Apart from the effects of ART, the direct association between adipose tissue and HIV results in a proinflammatory state and a disturbed metabolic and hormonal environment that increases the risk of CVD and other HIV-related non-communicable comorbidities [32].

HIV affects blood lipid levels, metabolism, and glucose homeostasis in addition to adipose tissue. HIV replication has a direct impact on host lipid metabolism. In a T cell line (in vitro), it induces cellular enzymes that enhance fatty acid synthesis, increase low-density lipoproteins (triglycerides), dysregulate lipid transport, oxidize lipids, and broadly alter lipid metabolic pathways [34]. Consistent with these mechanistic insights, higher HIV RNA levels were associated with lower LDL cholesterol and higher VLDL cholesterol and triglycerides. A history of AIDS-defining events was linked to higher total cholesterol, VLDL cholesterol, and triglyceride concentrations. Regarding glucose homeostasis, higher CD4+ counts were associated with less evidence of insulin resistance, suggesting that more advanced HIV disease is associated with less favorable lipid and glucose profiles [34,35]. Conversely, a Japanese cohort of men living with HIV (MHIV) patients who had not received treatment revealed a correlation between higher CD4^+^ counts and greater HDL- and LDL-C values [36]. Despite low LDL-C levels during the active replication phase, diminished HDL-C and elevated VLDL-C and triglycerides may elucidate the pro-atherogenic effects of HIV. So, HIV exemplifies a model disease for elucidating the roles of immunological activation and metabolic imbalance in the progression of CHD, MI, and HF [37].

## 3. Risk Factors of Common Cardiovascular Diseases in HIV

**Figure 3:**
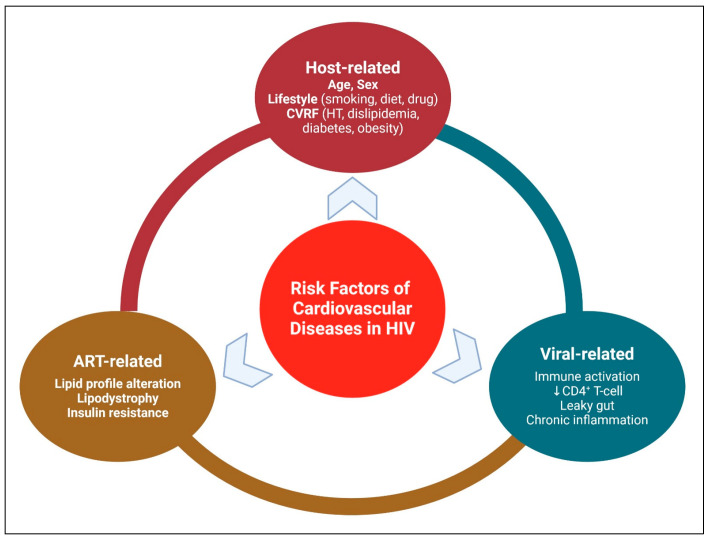
Risk Factors of Cardiovascular Diseases in HIV. Created in BioRender. Hetta, H. (2026) https://BioRender.com/ng596ew (accessed on 22 January 2026).

Common risk factors of CVD in HIV can be categorized into 3 main categories: Patient-related, viral-related, and ART-related (Figure 3).

### 3.1. Cardiovascular Risk Stratification in HIV-Positive Patients

Population studies have firmly demonstrated that there are sex variations in the risk pathways for CVD [38]. Furthermore, different sexes experience HIV infection differently in terms of how it causes immunological activation and/or metabolic dysregulation [39,40]. Together, these two variables suggest that there are sex disparities in both HIV-related CV risk and CVD risks among PWH. Analyzing sex-specific characteristics associated with HIV-associated CVD risk is crucial for the successful implementation of HIV-specific CVD prevention strategies for both sexes. Women living with HIV (WHIV) exhibit the highest level of systemic immune activation as compared to MHIV, non-HIV-positive males, and women living without HIV [40]. WHIV, compared to MHIV, typically exhibits unique patterns of metabolic dysfunction [41]. Concurrent general population research indicates that women exhibit greater susceptibility than men to heightened immunological reactivity and distinct patterns of metabolic instability. Consequently, a deeper understanding of sex-specific processes of CVD risk in WHIV may provide insights into similar mechanisms in women within the general population.

In high-income countries, sex-stratified analyses of myocardial infarction and coronary heart disease incidence indicate that women living with HIV may have a disproportionately higher risk of HIV-associated cardiovascular complications compared with men. Triant et al. studied data from the Partners Healthcare System in the United States (US) regarding MI rates among 1,044,598 control participants (59 percent female) and 3851 PWH individuals (30 percent female) who were followed up from 1996 to 2004 [42]. Concerning WHIV, MHIV, non-HIV-infected women, and non-HIV-infected men, the unadjusted incidence rates of MI per 1000 person-years were 12.71, 10.48, 4.88, and 11.44, respectively. After adjusting for conventional CVD risk variables, the RR of MI in WHIV compared to those without was 2.98 (95% Cl 2.33–3.75), and the associated risk for MHIV compared to those without was 1.40 (95% Cl 1.16–1.67) [42]. Sex-stratified analyses from a French cohort produced similar results, corroborating the heightened risk of HIV-related CHD and MI in women [43].

In a high-income country, sex disparities in MI subtype presentations in perinatally HIV-positive individuals are highlighted by recent research conducted by Crane et al. [44]. To determine the likelihood of any clinical MI, Crane’s team evaluated data from 26,909 PWH patients who underwent testing at one of six US medical sites between 1996 and 2014. 288 occurrences were classified as definite or probable type II MI, while 362 instances were classified as definite or likely type I MI throughout the cohort. The subtype distribution in WHIV who had MI was 46% (69/150) type I and 54% (81/150) type II, suggesting that type II was slightly more common. On the other hand, the subtype distribution of MHIV people who had MI was 41% (207/500) type II and 59% (293/500) type I, suggesting a minor predominance of type I. Although type II MI is associated with rates of major adverse cardiovascular events, cardiovascular death, and all-cause mortality that are comparable to those observed after type I MI, as reported in a large general population study by Gaggin et al. [45], its overall prognosis remains unfavorable, in part due to the absence of standardized, evidence-based treatment strategies. This highlights the urgent need for public health initiatives to develop preventative methods for high-risk populations. These strategies must be considered in an understanding of sex-specific and population-specific physiology.

### 3.2. HIV-Specific Risk Factors (e.g., Chronic Immune Activation, ART Side Effects)

HIV’s early attack on mucosal CD4+ T cells in the gastrointestinal tract disrupts the epithelial barrier, allowing bacteria and microbial products to translocate into the portal and systemic circulation, thereby generating persistent immune activation. Biomarkers such as soluble CD14 (sCD14) and lipopolysaccharide-binding protein (LBP) reflect the degree of microbial translocation and have been linked to increased risk of CVD. Longitudinal cohort studies have shown that elevated sCD14 and LBP levels correlate with endothelial dysfunction, subclinical atherosclerosis, and higher incidence of cardiovascular events, providing quantitative evidence of their predictive value in HIV-associated CVD risk assessment [46,47,48]. Growing data indicates that HIV is linked to the movement of bacteria and other microbiological products from the intestines into the bloodstream, giving support to this idea. Even in individuals with virological suppression, this situation prolongs endothelial damage, persistent immunological activation, and CVD [49,50,51,52]. In PWH with suppressed viremia, microbial translocation may provoke a distinct immune activation profile characterized by elevated levels of CD38(+) CD8(+) T cells and soluble thrombomodulin, a hallmark of endothelial activation [53]. Microbial translocation has been linked to an increased risk of thrombosis and hypercoagulability, in addition to ongoing immunological activation, which could lead to CVD. Studies revealed that macrophages and platelets in PWH often express tissue factor, an activator of the coagulation cascade; lipopolysaccharides and flagellin can stimulate monocytes to produce tissue factor [54].

Microbial dysbiosis and the movement of bacteria and microbial metabolites in PWH have been linked to several mechanisms. Initially, the virus both directly and indirectly targets circulating and mucosal CD4^+^ T cells by activating cytotoxic CD8^+^ T cells to destroy infected CD4^+^ T cells. Early in the course of an infection, the number of CD4^+^ mucosal T cells in the gastrointestinal tract decreases [50]. Secondly, HIV may impair tight connections between enterocytes, as previously documented in simian immunodeficiency virus infection [55]. These events erode the mucosal barrier of the gastrointestinal tract, allowing commensal bacteria and their components to enter the bloodstream. HIV also causes crypt hyperplasia, bacterial overgrowth, and villous atrophy, which all affect gastrointestinal lumen permeability [51]. Because PWH have fewer intestinal immunoglobulin (Ig)A-producing B-cells, their luminal IgA levels are lower, which puts them at risk for bacterial overgrowth and translocation [56].

### 3.3. Traditional Cardiovascular Risk Factors (e.g., Smoking, Hypertension)

Compared with the general population, people living with HIV have a higher prevalence of traditional cardiovascular disease risk factors, such as dyslipidemia, diabetes, smoking, and hypertension [57,58,59]. With a prevalence ranging from 4.8% to 73.4% in HIV patients, hypertension is a major risk factor for CVD [57]. Compared to Caucasians, African American PWH are more likely to have blood pressure regulation issues, which increases their vulnerability to adverse CV outcomes [60]. Endothelial function and integrity are altered by HIV-induced chronic inflammation, and the renin-angiotensin system is dysregulated in response to ART [61,62]. The prevalence of dyslipidemia among PWH ranges from 7.7% to 73.4%, with an average of 39.5% [57]. In this patient group, dyslipidemia may be caused by several mechanisms, such as hepatic de novo lipogenesis and increased basal lipolysis, reduced insulin efficiency in preventing adipocyte lipolysis, and impaired peripheral fatty acid trapping [63]. Moreover, PWH with diabetes mellitus or insulin resistance had a higher frequency of hyperlipidemia. Reduced HDL-C and high LDL-C are the markers of dyslipidemia in this population, with hypertriglyceridemia being the most common abnormality, particularly after ART initiation [64].

Older, rarely used ART might cause body fat to redistribute, showing up as either lipohypertrophy with increasing visceral fat or lipoatrophy in the face and limbs [65]. Because lipodystrophy is linked to hypertriglyceridemia, decreased HDL-C, dyslipidemia, increased insulin resistance, and diabetes mellitus, it is a substantial risk factor for CVD [65]. With a range of 0.5% to 39.1%, the average prevalence of diabetes mellitus is 7.24% [57]. It is associated with lipodystrophy, a low CD4^+^ count (<200 cells/μL), and the use of some older ART [66,67].

Smoking is prevalent among PWH, with a prevalence rate of 33% (ranging from 0% to 67%), resulting in significant cardiovascular morbidity and mortality. Furthermore, a significant correlation exists between illegal drug use and CVD in those living with HIV [57]. Heroin, marijuana, cocaine, and methamphetamine are the most often used illegal substances in this population [68]. All of these substances can cause arrhythmias and coronary atherosclerosis. A developing problem among MHIV who have sex with males is chemsex, which is described as the use of recreational drugs (including cocaine, ketamine, mephedrone, gamma-hydroxybutyrate/gamma-butyrolactone, and crystal methamphetamine) to enhance sexual experiences [69]. Although the CV implications of chemsex remain unexamined, this practice may negatively impact the CV system, particularly through the direct effects of these substances on cardiac function [70].

In addition to patient-related factors, emerging evidence suggests that genetic polymorphisms in inflammatory genes, such as IL-6 and TNF-α, may contribute to sex-specific disparities in CVD risk among WHIV. These variants can influence levels of systemic inflammation and immune activation, potentially exacerbating the cardiovascular impact of HIV and ART in this population. Although current data from genome-wide association studies (GWAS) in PWH remain limited, these findings highlight an important avenue for future research and suggest that integrating genetic risk factors could eventually support personalized risk stratification and targeted prevention strategies [71,72,73,74].

## 4. Impact of Antiretroviral Therapy on Cardiac Health

### 4.1. ART-Associated Risks

To summarize, ART has significantly increased the life expectancy of PWH; yet, it is not curative, is hazardous, and requires lifelong use. In addition to the clear and guaranteed advantages of modern ART, lifelong use presents specific hazards linked to cumulative long-term exposure to these medications. Cardiometabolic toxicity is one of the most prevalent and significant side effects. ART induces cardiometabolic toxicity by either exacerbating pre-existing CV risk factors or by introducing new ones (including dyslipidemia, insulin resistance, and weight gain) [75,76]. Furthermore, ART causes metabolic abnormalities first by altering lipid metabolism and, secondly, by directly causing tissue damage as a result of the actions of particular medications [36,77]. When compared to more recent treatments, older drugs—especially abacavir and first-generation protease inhibitors (PIs)—have been associated with a higher frequency of side effects [76,78]. PIs exacerbate hypertriglyceridemia by inhibiting lipoprotein lipase (LPL), the key enzyme responsible for hydrolyzing triglycerides in circulating lipoproteins. Mechanistically, PI-mediated LPL inhibition reduces triglyceride clearance, leading to elevated VLDL and chylomicron remnants. Kinetic modeling studies of lipid metabolism have quantified this effect, showing prolonged triglyceride residence time in plasma and increased atherogenic potential. In contrast, integrase strand transfer inhibitors (INSTIs) and newer NRTIs generally exert less adverse influence on lipid kinetics [79].

Early ART termination was linked to a higher risk of acute MI, per the Strategies for Management of Antiretroviral Therapy (SMART) trial. Most likely, rebound viremia-induced inflammation was the reason for this elevated risk [76,80]. However, the SMART trial demonstrated that in PWH with preserved immunity (CD4^+^ > 500 cells/mm^3^), early ART initiation was linked to distinct cardiometabolic outcomes; total cholesterol and LDL-C levels increased, while HDL-C levels increased and the need for antihypertensive medication decreased. These results suggested that early ART may not have a significant short-term impact on traditional risk variables [81]. The findings of the Vienna Central Hospital AIDS clinical data survey in Australia showed that after three months of medication with PIs, plasma cholesterol and triglyceride levels increased in HIV-positive patients. Triglyceride and LDL-C levels were significantly higher, whereas plasma HDL-C concentration was much lower [82,83].

#### Specific Classes of ART and Their Cardiovascular Effect

An international research team in Europe examined the association between NRTIs and CVD, with a focus on ART drug side effect data analysis, by following up on 157,921 HIV-positive patients over six years at 212 official AIDS medical facilities in Australia, the US, and other countries. When starting therapy with the NRTIs drugs abacavir and didanosine, the incidence of heart attacks increased by 49% and 90%, respectively, compared to HIV-positive patients who did not get these drugs [84,85]. The British Tropical Medicine Research Group also conducted a statistical analysis of clinical data that was gathered from the HIV Treatment Center Hospital in Limbe, Cameroon. Their research demonstrated a strong relationship between the use of ART drugs and the prevalence of hypertension in humans [86,87]. According to the statistical analysis, HIV-positive people who received ART had twice as high an incidence of hypertension as those who did not [88].

Patients with HIV frequently get cardiomyopathy, the most common type of cardiac illness. The etiology of AIDS-related cardiomyopathy remains unidentified. Numerous variables, including cytokine effects, HIV infection, inflammatory reactions, opportunistic infections, and the direct toxic effects of ART, especially NRTIs, can be linked to the loss of cardiomyocytes [89]. Cardiomyocytes have enough mitochondria and glycogen granules to maintain the heart’s constant, regular contractions. NRTIs medications have been found to induce mitochondrial dysfunction in cardiac tissue based on empirical findings from clinical and basic research [90]. Results showed that human cardiomyocytes exposed to low-concentration NRTI medications for an extended period showed decreased copy number and mitochondrial DNA depletion. Due to the identical architecture of mitochondrial DNA polymerase γ and HIV reverse transcriptase, NRTI drugs hinder HIV reverse transcriptase activity and mitochondrial DNA replication by inhibiting the activity of mitochondrial DNA polymerase γ in cells [91]. When mitochondrial DNA is removed, the peptides that it encodes are lost. These peptides are essential for oxidative phosphorylation and, in turn, cause mitochondrial dysfunction in cardiac cells [92]. Because of malfunctioning mitochondria, cardiomyocytes lose energy, their respiratory control ratio, and mitochondrial respiratory oxygen consumption decreases. Triglyceride accumulation and a decrease in the fatty acid oxidation ratio occur concurrently [93].

### 4.2. The Effect of ART on Heart Failure

The causes of HF are diverse in the general population and PWH. Before the advent of ART, HF was acknowledged as a consequence of advanced HIV, characterized by viremia, increasing immunological deterioration, widespread cardiac dysfunction, and frequently, inflammation. In addition, the presence of ART has broadened the causes and manifestations of HF in PWH. Individuals with chronic HIV viremia, immunological deterioration, and opportunistic infections may nonetheless develop HIV-associated cardiomyopathy characterized by significant systolic dysfunction, despite the absence of obstructive CHD [94]. Moreover, viremia and immunological progression contribute to subtle cardiomyopathy and HF, as both are linked to diastolic dysfunction in patients with HIV. However, the rising prevalence of CHD and MI in PWH has led to a growing incidence of ischemic heart failure, attributed to post-MI myocardial scarring and possible microvascular dysfunction. Toxic causes of myocardial dysfunction and HF significantly contribute to HIV-related HF due to the elevated prevalence of myocardial-toxic substance use (e.g., methamphetamines) among patients with HIV [94]. The side effects of the most important classes of ART are presented in Table 1.

## 5. Prevention and Management of Cardiovascular Diseases in HIV

Clinical data on CVD prevention strategies among HIV-positive people are essential in this context. Several studies conducted over the last 20 years have assessed the impact of statin treatment on several subclinical indicators of atherosclerosis and inflammation in HIV patients. The findings from these trials on vascular inflammation have been inconsistent. Nevertheless, statins seem to diminish specific inflammatory indicators and, as anticipated, lower atherogenic lipid levels (e.g., LDL-C) in PWH [94,99]. The effectiveness of statins in preventing severe atherosclerotic CHD endpoints in PWH is better understood thanks to the results of the randomized trial to prevent vascular events in HIV (REPRIEVE). Those with HIV who are at low to moderate risk of atherosclerotic CVD were randomized at random in REPRIEVE to receive a placebo or pitavastatin. 7500 PWH have been enrolled in this trial. The trial reported that the individuals had a median age of 50 years; the median CD4^+^ T cell count was 621 cells/mm^3^, and 5250 out of 5997 participants (87.5%) with available data had an HIV RNA result below quantitation. The REPRIEVE trial was stopped early following a recommendation by the Data and Safety Monitoring Board because pitavastatin significantly reduced the risk of major adverse cardiovascular events (MACEs) in PWH over a median follow-up of 5.1 years. 91 participants (2.3%) in the pitavastatin group and 53 participants (1.4%) in the placebo group reported experiencing muscle-related symptoms; 206 people (5.3%) and 155 participants (4.0%), respectively, reported having diabetes mellitus. This means HIV-infected participants administered pitavastatin exhibited a reduced risk of MACEs compared to those receiving a placebo, across a median follow-up period of 5.1 years [100].

The results of the REPRIEVE trial have had immediate and substantial implications for clinical practice. In contrast to earlier strategies that restricted statin therapy primarily to PWH at high estimated cardiovascular risk, the demonstrated reduction in MACEs with pitavastatin in a predominantly moderate- and low-risk population has prompted a paradigm shift toward broader primary prevention [100]. Accordingly, the updated European AIDS Clinical Society (EACS) guidance recommends considering statin therapy for primary prevention in PWH with moderate and low cardiovascular risk, reflecting the trial’s demonstrated benefit [101]. These updated recommendations highlight the importance of proactive cardiovascular prevention strategies in PWH, even in the absence of overt high-risk profiles.

In comparison to statins, there is even greater ambiguity over other CVD prevention strategies for PWH. Current studies, which are not yet sufficiently powered for clinical objectives, are assessing the efficacy of proprotein convertase subtilisin/kexin type 9 (PCSK9) inhibitors in patients with HIV. A prospective study aimed to assess the correlation between HIV-related dyslipidemia in PWH on protease inhibitors and PCSK9, a key regulator of LDL-C homeostasis. In addition to 90 HIV-negative controls who were age and sex-matched, 103 HIV-positive patients had their plasma PCSK9 levels measured both before and after initiating PI-based ART. ELISA was used to measure PCSK9. The results showed that after using protease inhibitors for a median of 14 months, PCSK9 levels in HIV-positive people did not increase and were consistently greater than in controls at all time points assessed (adjusted *p* value before and after: <0.05). Total cholesterol, LDL-C, and HDL-C levels increased after protease inhibitors were added; however, LDL-C levels stayed lower than those of the control group. When compared to immunodeficiency and the severity of HIV disease, PCSK9 levels showed a positive correlation at baseline, as demonstrated by the HIV-1 viral load (*p* = 0.01), CD4 T-cell count <200/μL (*p* = 0.002), and stage C HIV disease (*p* = 0.0002). Protease inhibitor-treated individuals showed a higher correlation between PCSK9 levels and glycemia, HDL-C, LDL-C, triglycerides, total cholesterol, and then with HIV-related factors. This suggests that HIV-positive individuals have higher PSCK9 levels. HIV may have an effect, as evidenced by the correlation between PCSK9 levels and infection severity in ART-naive patients. When PI-containing ART was initiated, PCSK9 levels associated with dyslipidemia in virologically suppressed individuals were comparable to those observed in controls [102].

HIV-positive people were not included in the 2020 American Society of Hematology (ASH) guidelines for antithrombotic and thrombolytic therapy. However, given the wealth of information regarding drug–drug interactions (DDIs) between ART and other medications, it is imperative to rigorously evaluate the possible interaction of antithrombotic therapies in PWH. PIs in particular, which make up the ART, have differing effects on the CYP450 liver enzymes that metabolize warfarin. As a result, careful INR monitoring is recommended. Warfarin DDIs vary, with some rising and others lowering levels. Individuals using lopinavir/ritonavir regimens need higher weekly warfarin dosages than those on efavirenz-based regimens, whereas HIV patients receiving therapy for tuberculosis need lower weekly warfarin dosages [103,104].

Recent evidence has explored the role of immune checkpoint inhibitors in populations historically excluded from major clinical trials, including PWH diagnosed with lung cancer [105]. There is a systematic review that synthesized data from multiple observational studies and case series to assess outcomes with programmed death-1 (PD-1) pathway blockade in this unique clinical context. The review demonstrated that PD-1 inhibitors, such as pembrolizumab and nivolumab, exhibit comparable efficacy in PWH with lung cancer to that reported in HIV-negative populations, with objective response rates and progression-free survival outcomes aligning with historical benchmarks. Importantly, the safety profile was acceptable, with immune-related adverse events occurring at rates similar to those seen in the general lung cancer population and no consistent evidence of HIV disease progression or significant increases in opportunistic infections during therapy. These findings support the feasibility and clinical utility of PD-1 inhibition in PWH with lung cancer while also underscoring the need for prospective studies to further define long-term outcomes and optimal management strategies in this under-studied group [106].

PIs and pharmacological enhancers that inhibit CYP 450 enzymes have the potential to increase the concentration of direct oral anticoagulants (DOACs) and increase the risk of bleeding. Treatment failure may result from the NNRTIs’ activation of CYP450 enzymes, which lowers the concentration of DOACs [107]. Careful selection should be used to manage interactions depending on patient characteristics; anticoagulants and ART with low risk of bleeding disorders should be used.

Additional anti-inflammatory treatments for patients with HIV are also important. These drugs aim to address gut health to diminish microbial translocation and intestinal inflammation, although they have not consistently influenced inflammation biomarkers in individuals with HIV. Additional therapies evaluated comprise canakinumab, an interleukin-1-beta (IL-1β) antagonist that diminished inflammatory markers and alleviated arterial and bone marrow inflammation in a limited study involving PWH. IL-1 primarily regulates a sequence of pro-inflammatory responses triggered by pathogen-induced tissue damage. Within the IL-1 family, IL-1β induces the overexpression of genes that enhance immune system activity and inflammatory response. Due to the increasing pathophysiological significance of IL-1β in various disease mechanisms, novel biological therapeutics have been discovered in recent years [108]. A drug that targets IL-1β, canakinumab, was recently approved for use in clinical settings. In this regard, the most recent results from the CANTOS trial are encouraging. The results show that, as compared to a placebo, anti-inflammatory treatment with canakinumab at a dosage of 150 mg given every three months significantly decreased the risk of recurrent CV events. These drugs’ ability to decrease cholesterol did not affect the outcomes. If the CANTOS trial’s findings were broadly applicable, they would support the idea that inflammation is the primary cause of atherothrombosis, which would mean that cytokine-targeted therapy is essential for secondary CVD prevention. Moreover, the potential benefits of canakinumab’s exceptional suppression of the inflammatory cascade must be carefully balanced against the drug’s unclear long-term safety profile [109].

Furthermore, methotrexate (MTX) marginally lowered CD8^+^ T cell counts in HIV-positive patients but did not affect inflammatory markers [110]. Recently, 176 HIV-positive patients on ART completed a low-dose methotrexate (LDMTX) randomized, double-blind, placebo-controlled trial conducted by the AIDS Clinical Trials Group (ACTG) to look at the drug’s anti-inflammatory and perhaps cardioprotective effects. The plasma levels of C-reactive protein (CRP), IL-6, IP-10, d-dimers, CD14, CD163, and fibrinogen did not show any significant changes in response to LDMTX; the only significant change observed was a modest drop in the levels of vascular cell adhesion molecule (VCAM). Significant drops in circulating CD4^+^ and CD8^+^ T cell counts were the main immunologic effects of LDMTX [111]. The principal mode of action of these widely used anti-inflammatory medications in PWH is determined by an experimental study. In trial samples, T-cell morphology and other plasma inflammatory markers were assessed. MTX reduced cycling (Ki67+) T cells lacking Bcl-2, whereas plasma inflammatory cytokines stayed essentially unchanged. Through a series of in vitro experiments designed to clarify the mechanisms underlying MTX activity, it was found that MTX inhibited mitochondrial function, T cell proliferation following mechanistic targets of rapamycin (mTOR) activation, and cell cycle entrance rather than effector cytokine production. Daily folic acid administration did not lessen the inhibitory effect in trial participants. Given that the inhibitory effect was reversible with folinic acid, it appears that low-dose MTX predominantly inhibits T cell proliferation in vivo in PWH by dihydrofolate reductase inhibition [112].

In the absence of compelling facts to the contrary, it is recommended to employ risk-based strategies for CVD preventive therapy in PWH, acknowledging that as CVD risk escalates, the absolute and net benefits of statin therapy for CVD prevention also increase. Additionally, it is generally acknowledged that the early beginning of ART aids in managing dyslipidemia alongside the suppression of HIV viremia.

## 6. Clinical Guidelines and Recommendations on Managing Cardiovascular Risk in HIV Patients

Cardiovascular risk assessment is a cornerstone of preventive care in people with HIV (PWH). Current guidelines recommend estimating the 10-year risk of atherosclerotic cardiovascular disease (ASCVD) using validated tools such as the ACC/AHA ASCVD Risk Calculator, as outlined in the 2013 ACC/AHA cardiovascular risk assessment guidelines and the 2018 updates on cholesterol and blood pressure management [113,114]. Cholesterol-lowering therapy is indicated for individuals aged ≥21 years with established ASCVD or LDL-C ≥190 mg/dL, for adults aged 40–75 years with diabetes mellitus regardless of calculated risk, and for those with a 10-year ASCVD risk ≥7.5%.

Given that traditional risk scores tend to underestimate cardiovascular risk in PWH, the American Heart Association recommends adjusting calculated risk upward by approximately 1.5–2-fold to account for HIV-related factors. Alternative tools include the Framingham 10-year CVD risk score (treatment threshold ≥10%) and the HIV-specific D:A:D model (5-year risk ≥3.5%) [110]. More recently, the 2021 ESC/EAS guidelines introduced the SCORE2 and SCORE2-OP models to estimate fatal and nonfatal cardiovascular risk in individuals below and above 70 years of age, respectively, and highlight the increased susceptibility of PWH to coronary and peripheral vascular disease, particularly in those with CD4^+^ counts below 200 cells/mm^3^ [115].

Lipid management in PWH should follow ESC/EAS recommendations. Statin therapy is advised for patients with dyslipidemia to achieve LDL-C levels <70 mg/dL with at least a 50% reduction from baseline in high-risk individuals, while more stringent targets (<55 mg/dL) are recommended for those at very high risk or requiring secondary prevention [116]. The European AIDS Clinical Society (EACS) 2021 guidelines further recommend annual cardiovascular risk assessment using Framingham or D:A:D scores in men over 40 years and women over 50 years. In PWH without established CVD but with a 10-year risk ≥10%, modification of antiretroviral therapy (ART) should be considered to avoid agents associated with increased cardiovascular risk, such as zidovudine or abacavir [117].

Optimization of ART is a key strategy for reducing cardiovascular risk. Evidence from large cohorts, including the SMART and D:A:D studies, indicates that certain antiretroviral agents—particularly protease inhibitors such as lopinavir/ritonavir and darunavir, as well as abacavir and didanosine—are associated with an increased risk of myocardial infarction and stroke [80,98]. In patients at elevated cardiovascular risk, switching to regimens with more favorable cardiometabolic profiles, such as integrase strand transfer inhibitors (INSTIs) and tenofovir alafenamide (TAF), is recommended. The REPRIEVE trial (2023) further underscored the benefit of statin therapy in PWH receiving ART, supporting a combined approach of ART optimization and lipid-lowering therapy for effective cardiovascular prevention [100].

Beyond pharmacologic interventions, cardiovascular risk reduction in PWH requires a comprehensive, multifaceted approach. Lifestyle modification remains foundational and includes smoking cessation, adherence to a heart-healthy diet, regular physical activity, and weight management. These measures reduce traditional cardiovascular risk factors while also improving metabolic and immune health [118]. Equally important is the systematic management of comorbidities such as hypertension, diabetes mellitus, and metabolic syndrome, which are highly prevalent in PWH and substantially amplify cardiovascular risk. Regular monitoring and early, aggressive treatment of blood pressure, glycemic control, and lipid abnormalities are essential to prevent long-term cardiovascular complications [119].

Finally, statins play an increasingly important role in primary cardiovascular prevention in PWH. Their use should be guided by individualized risk assessment using tools such as the ASCVD calculator, Framingham score, or D:A:D model, and informed by emerging evidence, including findings from the REPRIEVE trial [100]. In the presence of HIV-specific risk factors, statin therapy may be justified even in individuals classified as having moderate or lower traditional risk, with careful attention to LDL-C targets and potential drug–drug interactions with ART [118,119].

Overall, this integrated approach—combining accurate risk stratification, lifestyle modification, ART optimization, comorbidity management, and appropriate statin therapy—provides a structured framework for improving long-term cardiovascular outcomes in people living with HIV.

## 7. Future Research Directions

The care of CVD is still controversial. HIV is considered a significant CVD risk factor, so future research should focus on certain elements of the diagnosis and management of CVD in PWH. This includes the investigation of biomarkers potentially linked to early diagnosis and prognosis, alongside innovative therapies aimed at addressing sustained immune activation and inflammation.

## 8. Conclusions

HIV infection is intricately linked to an increased risk of CVD, stemming from a combination of chronic inflammation, immune activation, traditional risk factors, and ART-related metabolic changes. The pathophysiology of HIV-associated CVD is multifaceted. The prevention strategies, including optimizing ART regimens to minimize cardiovascular side effects, are critical in reducing the CVD burden in PWH. Current clinical guidelines emphasize early screening for CVD by using biomarkers and aggressive management tailored to the unique needs of PWH. However, further research is required to refine prevention strategies. Future directions should focus on personalized cardiovascular risk management in PWH, incorporating genetic, environmental, and ART-related factors, along with exploring novel therapies targeting inflammation and immune activation.

## Figures and Tables

**Table 1:** The cardiotoxicity effects of the most important classes of antiretroviral therapy. Abbreviations: MI: myocardial infarction, HF: heart failure, CVD: cardiovascular diseases, CHD: coronary heart disease.

Drug Class	Drug Name	Side Effects	References
Nucleoside/nucleotide reverse transcriptase inhibitors (NRTIs)	Didanosine (ddi)	Increased risk of MI and cardiomyopathy.	[95]
	Zidovudine (AZT)	Cardiomyopathy, HF, and left ventricular dysfunction.	[96]
	Abacavir (ABC)	Increased risk of MI (controversial but observed in some studies).	[95]
	Lamivudine (3TC)	Limited direct cardiac toxicity data is considered relatively safe in terms of cardiotoxicity.	[96]
	Tenofovir disoproxil fumarate (TDF)	Potential for cardiovascular side effects due to bone and renal issues indirectly affecting cardiac function	[97]
	Tenofovir alafenamide (TAF)	Lower cardiovascular risk compared to TDF, better renal and bone safety profile.	[97]
Protease inhibitors (PIs)	Ritonavir (RTV)	QT prolongation, PR interval prolongation, bradyarrhythmia, increased risk of MI.	[12,78]
	Lopinavir/ritonavir (LPV/r)	Increased risk of MI and dyslipidemia leading to CVD.	[82]
	Atazanavir (ATV)	PR interval prolongation, less dyslipidemia compared to other PIs but still associated with cardiovascular events.	[82]
	Darunavir (DRV)	Associated with MI risk and dyslipidemia.	[98]
	Saquinavir (SQV)	QT prolongation, PR interval prolongation, and risk of arrhythmias.	[12,78]
	Indinavir (IDV)	Associated with endothelial dysfunction and CHD.	[12,78]

## Data Availability

No new data were created or analyzed in this study. Data sharing is not applicable to this article.
